# MicroRNAs regulating apoptosis and delivery strategies for treating intervertebral disc degeneration

**DOI:** 10.3389/fcell.2025.1683247

**Published:** 2025-10-28

**Authors:** Yuwei Chen, Chuan Guo, Weiqiang Lan, Yuheng Liu, Fei Ma, Daqiang Zheng, Xueyuan Xu, Qingquan Kong, Yu Wang

**Affiliations:** ^1^ Department of Orthopedic Surgery and Orthopedic Research Institute, West China Hospital, Sichuan University, Chengdu, Sichuan, China; ^2^ West China School of Medicine, West China Hospital, Sichuan University, Chengdu, Sichuan, China; ^3^ Department of Urology and Institute of Urology (Laboratory of Reconstructive Urology), West China Hospital, Sichuan University, Chengdu, Sichuan, China

**Keywords:** intervertebral disc degeneration, nucleus pulposus cell, apoptosis, microRNA, delivery strategies

## Abstract

Intervertebral disc degeneration (IDD) is a leading contributor to chronic low back pain (LBP), significantly impairing quality of life and imposing socioeconomic burdens globally. Current treatments fail to address the underlying pathology or restore intervertebral disc (IVD) function. IDD is multifactorial but strongly linked to nucleus pulposus cell (NPC) apoptosis. Gene therapy, particularly the use of microRNAs (miRNAs), holds promise for reversing IDD by targeting apoptotic pathways and enhancing the biological functions of NPCs. Given the avascular nature of IVD, effective delivery strategies are critical. Targeting strategies, such as NPC-targeting, which relies on cellular uptake through direct uptake, specific ligand‒receptor interactions and aptamer utilization, can significantly increase delivery efficacy. Additionally, organelle-targeting strategies that focus on mitochondria and the endoplasmic reticulum (ER) optimize therapeutic delivery. Coupled with these targeting methods, responsive delivery strategies that react to endogenous stimuli (such as pH, reactive oxygen species, and enzymes) and exogenous stimuli (including temperature, ultrasound, magnetism, and light) enable spatiotemporal release of therapeutic agents. This review summarizes the role of anti-apoptotic miRNAs in regulating NPC apoptosis and the latest advances in targeting and responsive delivery strategies to overcome the challenges associated with targeted therapeutic miRNA application, offering new insights into the treatment of IDD.

## 1 Introduction

Low back pain (LBP) causes more disability than any other diseases worldwide, and its prevalence has significantly escalated since 1999 (Hoy et al., 2014; Ritchie, 1984). A staggering 619 million people worldwide suffered from low back pain in 2020, and its prevalence is projected to increase (The Lancet, 2023). Although LBP is considered multifactorial and complicated, intervertebral disc degeneration (IDD) is one of the major causes of LBP, accounting for 40% of cases (Hoy et al., 2015; Zhu et al., 2024). IDD is a very common chronic, prevalent and age-related degenerative musculoskeletal disorder that significantly impairs function and quality of life compared with other diseases, thus leading to an enormous socioeconomic burden worldwide (Luckhaupt et al., 2019; Moradi-Lakeh et al., 2017).

The intervertebral disc (IVD) is crucial for spinal biomechanics but is prone to degeneration. The process of IDD is an aberrant, cell-mediated response to progressive structural failure that is inextricably associated with the death of IVD cells. IDD initiates NPC apoptosis and progressively affects adjacent tissues (Vergroesen et al., 2015). Since the apoptosis of NPCs plays a crucial role in IDD, a better understanding of their intrinsic and extrinsic signaling pathways may lead to the exploration of promising potential therapeutic targets. Current clinical therapeutic methods have many limitations and cannot reverse the IDD. Conservative treatment can alleviate only clinical symptoms, and most patients ultimately require surgical intervention (Gugliotta et al., 2016). Neither minimally invasive surgery nor traditional surgery can overcome the risks of surgical injury, postoperative recurrence, accelerated degeneration, uncertain long-term efficacy, and other problems (Xie et al., 2020). Furthermore, no current therapeutic approach directly targets the molecular mechanisms driving IDD progression, leaving an urgent need for innovative and effective treatment strategies to alleviate patient suffering and reduce the socioeconomic burden. Gene therapy can reverse the process of IDD by inducing changes in intradiscal gene expression, and therapeutic genes are specifically designed and delivered to specific target sites. MicroRNAs (MiRNAs), short noncoding RNAs that regulate gene expression, originating from pri-miRNA transcription, has shown great potential for disease therapy (Wang T. J. et al., 2024). Different kinds of miRNAs are involved in extracellular matrix (ECM) degeneration, apoptosis, inflammation and mechanobiology, indicating that miRNAs play important roles in the pathology of IDD (Ohrt-Nissen et al., 2013). Recent breakthroughs in miRNA-based gene therapy, including the anti-apoptotic effects and the stronger effects result from the interaction with other molecules in the pathway, demonstrate their significant therapeutic potential in reversing IDD progression and restoring disc integrity.

As the IVD is an avascular enclosed organ with a harsh microenvironment, it is difficult to achieve therapeutic concentrations of bioactive agents via systemic administration. Therefore, the NPC-targeting or organelle-targeting delivery strategy of miRNA can either achieve the ideal efficacy or reduce the potential toxicity and waste of dosage caused by random distribution. Many studies have explored spatiotemporal controlled release strategies for gene delivery, which aim to respond and adapt to the microenvironment and achieve the desired therapeutic effect (Guo et al., 2024; Wang Y. et al., 2023). The progression of IDD is accompanied and promoted by increased expression of endogenous signaling molecules, such as degenerative-related enzymes, reactive oxygen species (ROS), and acidic pH. Due to the simultaneous changes between therapeutic targets and responsive factors, developing a responsive delivery system holds great promise. Some exogenous signals can also reach responsive delivery, including temperature, light, ultrasound, and magnetism. On the basis of a certain level, adjusting the dosage appropriately can be used as a signal. Since the direct delivery of miRNAs has many limitations, novel biomaterials aimed at achieving the desired therapeutic efficacy have been synthesized (Kang et al., 2023). This review summarizes the apoptosis of NPCs, potential anti-apoptotic miRNAs used in IDD gene therapy, targeting and responsive delivery strategies aimed at slowing and reversing the progression to attenuate IDD.

## 2 Apoptosis of the NPC

### 2.1 NPC function in IDD

The nucleus pulposus (NP), the core of the IVD, contains randomly organized collagen fibers and radially arranged elastin fibers embedded in a highly hydrated aggrecan-containing gel and is sandwiched inferiorly and superiorly by cartilage (Raj, 2008). NP contains approximately 3,000 cells/mm^3^ and is composed of notochordal cells derived from the embryonic notochord and chondrocyte-like cells resembling the phenotype of articular chondrocytes but expressing specific markers (Frapin et al., 2019; Risbud et al., 2015; Clouet et al., 2009). These two cell types initially coexist until skeletal maturity in humans, after which notochordal cells gradually differentiate into chondrocyte-like cells with aging. NPCs are essential for preserving intervertebral disc integrity by producing key components of the extracellular matrix (ECM), such as aggrecan and collagen types I, II, and X. These components help maintain the osmotic pressure critical for the biomechanical properties of the NP (Loreto et al., 2011). There is a cellular interaction between these two cell types that regulates the secretion of cytokines, limiting the enzymatic degradation of the ECM, protecting NPCs from apoptosis and stimulating the synthesis of ECM components (Erwin et al., 2006). Taken together, the survival and activity of NPCs and their interactions indicate the normal status of IVD. The initial degeneration of IVD is mostly embodied in the dysfunction of NPCs. Therefore, enhancing the biological function of NP cells will be a pivotal direction for designing regenerative treatment methods.

### 2.2 Mechanisms of NPC apoptosis in IDD

Since the NP provides support for the IVD and ensures the flexibility of the spine, NPCs may be exposed to many unfavorable factors that induce the apoptosis of NPCs, thus leading to a series of pathological changes, resulting in IDD (Li et al., 2023). These harmful factors disrupt signaling pathways by acting on various signaling molecules and interacting with each other to cause NPC apoptosis. NPCs are referred to as type II apoptotic cells and undergo caspase-independent apoptosis via two independent but associated pathways: the intrinsic and extrinsic pathways (Ozören and El-Deiry, 2002). Currently, the widely studied pathways mainly include the mitochondrial signaling pathway and the endoplasmic reticulum (ER) signaling pathway in the intrinsic pathway and the death receptor (DR) signaling pathway in the extrinsic pathway.

#### 2.2.1 Mitochondrial signaling pathway

**FIGURE 1:**
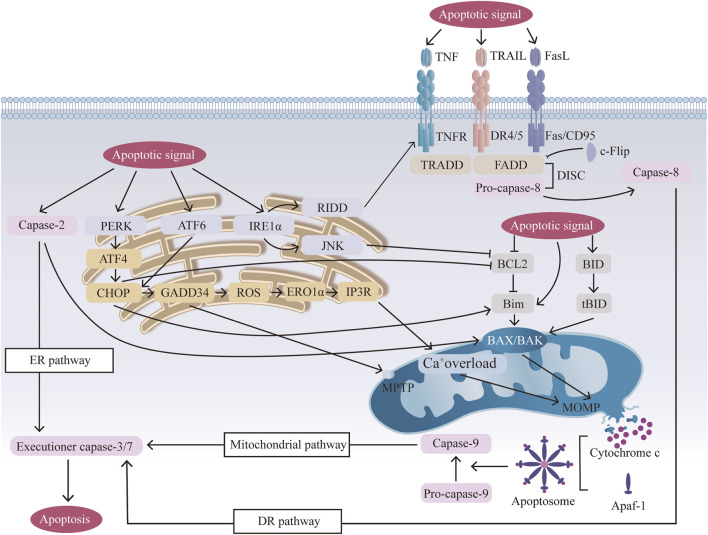
Mechanisms of three apoptotic pathways: the mitochondrial pathway, the (endoplasmic reticulum) ER pathway, and the (death receptor) DR pathway. Mitochondrial pathway: apoptotic signals activate BCL2 family members, and activated BAX/BAK undergoes oligomerization, forming BAX/BAK pores in the mitochondrial membrane, releasing CYCS, and then forming apoptosomes, which activate caspase 9 and induce apoptosis. Mitochondrial Ca+ overload and mitochondrial permeability transition pore (MPTP) opening further amplify this signal. ER pathway: ER stress from cellular stressors activates sensors (activating transcription factor 6 (ATF6), inositol-requiring enzyme (1αIRE1α), and protein kinase RNA-like ER kinase (PERK)), leading to C/EBP-homologous protein (CHOP) activation, mitochondrial dysfunction, and apoptosis. CHOP mediates ER-mitochondrial crosstalk by suppressing BCL2 and activating BIM, thereby forming a positive feedback loop that amplifies BAX/BAK oligomerization. ER stress also causes calcium release, contributing to mitochondrial calcium overload and apoptosis. DR pathway: Activation of death receptors (Fas, TNF-related apoptosis-inducing ligand (TRAIL) receptors, DR4/5) by their ligands (FasL, TRAIL, TNF) results in the formation of the death-inducing signaling complex (DISC), which activates caspase 8 and triggers apoptosis via direct cleavage of downstream executioner caspases. BH3-interacting domain death agonist (BID) cleavage amplifies this pathway by activating BAX/BAK and the mitochondrial pathway.

The mitochondrial signaling pathway is induced by various cellular stresses and numerous apoptotic signals, such as oxidative stress, inflammation, and mechanical stress (Ding et al., 2012; Feng et al., 2017; Mehrkens et al., 2017), triggering changes in mitochondrial outer membrane permeabilization (MOMP). The BCL2 family plays a key role in regulating mitochondrial apoptosis by controlling MOMP. In response to apoptotic signals, proapoptotic members (BCL2 associated X, apoptosis regulator [BAX], and BCL2 antagonist/killer 1 [BAK1, also known as BAK]) are activated and undergo oligomerization to form BAX/BAK-lipid pores in the mitochondrial outer membrane, whereas anti-apoptotic members (BCL2 and BCL2 like 1 [BCL2L1, also known as BCL-XL]) are inhibited. BCL2 and BCL-XL can bind to the BH3-binding pocket and interact with the BCL2-interacting mediator of cell death (Bim), thereby preventing Bim from activating Bax/Bak (Singh et al., 2019). The stimuli may also lead to the cleavage of BH3-interacting domain death agonist (BID) to form the active, truncated form tBID. tBID can further translocate to mitochondria and change MOMP through the activation of BAX and BAK1 (Letai et al., 2002). BAK is constitutively mitochondrial, but BAX is cytosolic and inserts into the membrane only upon activation (Chipuk et al., 2006). Many mitochondrial proteins, including mitochondrial cytochrome c (CYCS), second mitochondria activator of caspase (Smac), apoptosis-inducing factor (AIF), endonuclease (Endo G) and serine protease (OMI, also known as HTR2A), are subsequently released from the mitochondrial space to the cytoplasm (Maas et al., 2010). Released CYCS combines with apoptotic protease activating factor-1 (Aparf-1) and caspase-9 to form apoptosomes (Marsden et al., 2002). Moreover, the overload of Ca^+^ leads to the loss of the mitochondrial membrane potential (ΔΨm), triggering the opening of the mitochondrial permeability transition pore (MPTP), which can also lead to release (Yao et al., 2024). Eventually, caspase-9 induces the activation of executioner caspases (caspase-3, caspase-6 and caspase-7) to trigger a cascade of caspases, such as cleavage and subcellular destruction, leading to apoptosis (McIlwain et al., 2015; Figure 1).

#### 2.2.2 ER signaling pathway

The ER is an important membranous organelle for protein synthesis, folding and secretion. When the folding function of ER proteins is disrupted by stimuli such as oxidative stress, imbalanced calcium homeostasis, inflammation, excessive mechanical loading and metabolic disorders (Dai et al., 2024), many unfolded or misfolded proteins accumulate in the ER lumen, and a series of subsequent reactions called endoplasmic reticulum stress (ERS) occur (Sakai and Grad, 2015). The early unfolded protein response (UPR) involves the activation of three signaling pathways by three biosensors (activating transcription factor 6 (ATF6), inositol-requiring enzyme (1αIRE1α), and protein kinase RNA-like ER kinase (PERK)) on the endoplasmic reticulum membrane to cope with damage (Seo et al., 2010; Ron and Hubbard, 2008). As the duration and intensity of stress increase above a certain threshold, adaptive ability becomes inadequate, and unresolved ER stress results in apoptosis (Tabas and Ron, 2011). Prolonged PERK signaling increases the expression of the proapoptotic transcription factor C/EBP-homologous protein (CHOP), which is mediated by the continuous activation of ATF4. CHOP is closely connected to mitochondrial apoptosis through the upregulation of proapoptotic BCL-2 family members and the downregulation of antiapoptotic BCL-2 family members to trigger MOMP (Szegezdi et al., 2006). CHOP also induces the expression of growth arrest and DNA damage-inducible 34 (GADD34), which causes ROS translation (Ma and Hendershot, 2003), upregulates ER oxidase 1α (ERO1α), and then activates inositol-1,4,5 trisphosphate receptor (IP3R), thus leading to the oxidation of the ER lumen and an increase in mitochondrial calcium (Li et al., 2009). Both IP3R and GADD34 contribute to the opening of the MPTP, which promotes apoptosis (Li et al., 2009; Oyadomari and Mori, 2004). Active IRE1α tends to improve the sensitivity of NPCs to apoptosis by activating IRE1α–JUN N-terminal kinase (JNK), which upregulates BAX expression and downregulates antiapoptotic BCL-2 expression, initiating mitochondrial apoptosis (Hetz, 2012)^,^ and IRE1-dependent decay (RIDD), which encodes chaperones and activates death receptor 5 to promote apoptosis through the extrinsic pathway (Urra et al., 2018). Caspase 2 may also participate in ER stress-mediated apoptosis by activating BAK and BAX and as an initiator to stimulate the caspase cascade (Upton et al., 2008; Figure 1).

#### 2.2.3 DR signaling pathway

The DR signaling pathway is the crucial extrinsic pathway triggered by various external stimuli leading to apoptosis. The death receptor pathway is activated by the interaction of specific ligands with death receptors, and three main signaling pathways are activated by these receptors: tumor necrosis factor (TNF), Fas/CD95 and TNF-related apoptosis-inducing ligand (TRAIL) receptors (Ashkenazi and Dixit, 1998). The Fas pathway occurs when the Fas ligand binds to the death domain (DD) of the Fas trimer. The activated Fas receptor then recruits the adaptor protein Fas-associated death domain (FADD), which consists of two interaction domains: a death domain (DD) and a death effector (DED) (Peter et al., 2007). After DDs interact with the Fas receptor, FADD binds to pro-caspase 8 through DED, which together form a complex protein called the death-inducing signaling complex (DISC) (Seo et al., 2018). FADD also recruits c-FLIP, which is an inhibitor that shares a similar structure to that of caspase 8 with no protease activity and competes with caspase-8 for binding to the DISC (Micheau et al., 2002). Then, through the DISC, pro-caspase 8 undergoes autocleavage to form caspase 8, which activates the effector protein caspase 3, generating a cascade of caspases that ultimately leads to apoptosis (Thorburn, 2004). The TRAILR pathway is related to the Fas pathway after the initial process in which DR4 and DR5 bind to the ligand TRAIL and then combine with FADD, recruiting pro-caspase 8 to form the DISC and activate caspase 3, thus causing apoptosis (Zhang X. B. et al., 2021). The TRAILR pathway and the TNF pathway are related to the Fas pathway after the initial process in which TRAILR binds to the ligand TRAIL and TNFR binds to the ligand TNF, respectively. However, for the TNF pathway, after TNFR binds to the ligand TNF, the activated receptor recruits the adaptor protein TNF receptor-associated death domain protein (TRADD), which then recruits FADD (Chen Y. et al., 2022). In addition to the formation of protein complexes that lead to the activation of caspases, there is another mechanism by which the death receptor signal is amplified by the mitochondrial signaling pathway. Bid is cleaved by caspase 8 and moves to the mitochondria to activate the intrinsic pathway, which not only connects the two caspase activation pathways but also amplifies the death receptor signal (Wang H. et al., 2011; Figure 1).

### 2.3 NPC apoptosis in IDD

The role of NPC apoptosis in IDD can be divided into three parts: the regulation of other types of cell death, inflammation, and ECM degradation. Apoptosis and other forms of programmed cell death are intricately interconnected and regulated, particularly in complex and challenging IDD environment. The major type of cell death varies during different periods of IDD, and the molecular mechanisms underlying the simultaneous occurrence of IDD may be explained by PANoptosis, which consists of pyroptosis and apoptosis (Li et al., 2022). A study has found the key genes such as NLRP3, GSDMD, and AIM2 regulate the PANoptosis. Among them, GSDMD deficiency causes NPCs to lean toward apoptosis, while activation of the NLRP3 inflammasome can simultaneously trigger caspase-1-mediated pyroptosis and caspase-3-dependent apoptosis. Infiltration of immune cells releases pro-inflammatory factors to exacerbate the synergistic activation of the two, forming a vicious cycle of inflammation-cell death-extracellular matrix degradation that jointly causes the progression of IDD (Zhou et al., 2024). However, there are few studies on PANoptosis in IDD, and more research is needed to deeply explore this area. In response to stimulation-induced apoptosis of NPCs in IDD, the activation of autophagy can inhibit apoptosis and help maintain intracellular homeostasis. Zhou. W et al. used 3-methyladenine (3-MA), an inhibitor of the early stage of autophagy, to block autophagy and reported that 3-MA could attenuate the protective effect of FNDC5/irisin on apoptosis, indicating that FNDC5/irisin suppresses apoptosis through activating autophagy (Zhou W. et al., 2022). Luo. L et al. also reported that cartilage endplate stem cell-derived exosomes inhibited NPC apoptosis and attenuated IDD in rats via activation of the AKT and autophagy pathways (Luo et al., 2021). A study revealed that LPS-induced NPC apoptosis was further increased by pretreatment with the autophagy inhibitor chloroquine (CQ), demonstrating that apoptosis and autophagy are antagonistic to each other (Yi et al., 2019). Moreover, characterized by a typical mode of inflammatory cell death, apoptosis unleashes proinflammatory factors in IVD cells either directly or indirectly, triggering inflammation (Jimbo et al., 2005). The excessive apoptosis of NPCs, which produce cartilage-specific ECM components, is an evident cellular and biochemical change that occurs during IDD (Gruber and Hanley, 2003). ECM degradative molecules are increasingly secreted during the process of apoptosis by NPCs (Vo et al., 2013). The dynamic balance between ECM synthesis and degradation is disrupted, resulting in a gradual loss of disc ECM, structural failure and biomechanical changes. The excessive loss of NPCs due to apoptosis disrupts ECM homeostasis, which is closely related to ECM degradation (Zhang Q. C. et al., 2021).

Both the intrinsic and extrinsic pathways of NPC apoptosis have been studied and proven to be closely linked to IDD (Wang H. et al., 2011). Park. et al. reported that Fas mediated apoptosis through the activation of two initiator caspases, caspase-8 and caspase-9, as well as the executioner caspase, caspase-3. These findings suggest that the apoptosis of NPCs involves both the DR pathway and the mitochondrial signaling pathway, with the Fas receptor playing a central role (Park et al., 2008). Liu. J et al. reported that the expression of BNIP3, a member of the BH3-only subfamily of Bcl-2 proteins, increased during cell apoptosis, suggesting a potential link between BNIP3 and the mitochondrial signaling pathway involved in NPC apoptosis (Liu et al., 2012). Ding. F et al. revealed that prolonged exposure to compression induced the mitochondrial signaling pathway via the activation of caspase-3 and Bax and the inhibition of Bcl-2 (Ding et al., 2012). Xie. Z Y et al. reported that ER stress was the key to attenuating acid-induced NPC apoptosis via the downregulation of CHOP and caspase12, demonstrating that the ER pathway is involved in IDD (Xie et al., 2017). Generally, the intrinsic and extrinsic pathways of NPC apoptosis play dominant roles in different stages of IDD. As Wang. H et al. reported that, when biomarkers of these three signaling pathways are detected, the DR signaling pathway is predominant in mild and moderate IDD, the mitochondria signaling pathway is predominant in moderate and severe IDD, and the ER pathway is predominant in mild IDD (Wang H. et al., 2011).

## 3 MiRNAs regulating apoptosis in nucleus pulposus cells

**FIGURE 2:**
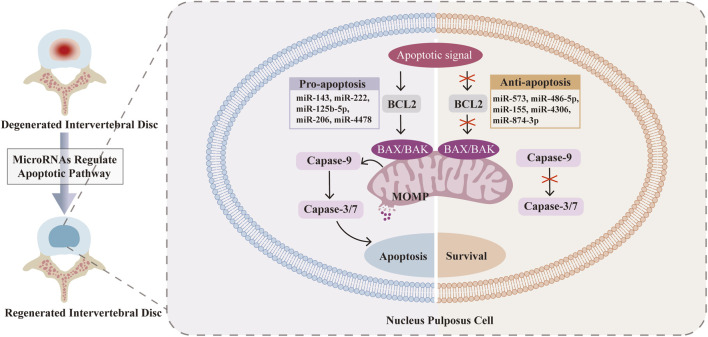
MicroRNAs (MiRNAs) in the regulation of key molecular interactions of nucleus pulposus cells (NPCs) apoptosis in Intervertebral disc degeneration (IDD). The upregulation of miR-143 (Zhao et al., 2017), miR-222 (Wang W. et al., 2019), miR-125b-5p (Jie et al., 2021), miR-206 (Zhou P. et al., 2022) and miR-4478 (Zhang J. et al., 2023) promotes the apoptosis of NPCs. The upregulation of miR-573 (Wang R. et al., 2019; Lin et al., 2021), miR-486-5p (Chai et al., 2019), miR-155 (Qin et al., 2023), miR-4306 (Wang D. et al., 2023) and miR-874-3p (Wang X. et al., 2024) inhibits the apoptosis of NPCs.

MiRNAs are small, noncoding RNA molecules that regulate gene expression and intracellular processes by binding to the 3′-untranslated region (3′-UTR) of target mRNAs, inhibiting protein translation and promoting mRNA cleavage. Therefore, the dysregulation of miRNAs is associated with various pathologies, including IDD, which makes it possible to use specific miRNAs to influence the initiation and progression of IDD (Jung et al., 2012; Ji et al., 2018). In the process of IDD, certain miRNAs can be upregulated or downregulated, varying in accordance with IDD progression (Table 1). Many miRNAs have been proven to regulate the intrinsic and extrinsic pathways of NPC apoptosis. In the mitochondrial pathway, miR-143 (Zhao et al., 2017) and miR-222 (Wang W. et al., 2019) upregulate the expression of BCL2, miR-573 (Wang R. et al., 2019; Lin et al., 2021) downregulates the expression of bax, and miR-145 (Zhou et al., 2019) downregulates the expression of ADAM17. In the DR pathway, miR-155 (Wang H. Q. et al., 2011), miR-499a-5p (Sun et al., 2019), miR-25-3p (Zhao et al., 2020) and miR-532-5p (Zhu et al., 2020) downregulate the expression of FADD, caspase-3, SOX4, bim and RASSF5, respectively (Figure 2). Rooted in these findings, miRNA inhibition therapy and miRNA restoration strategies have been proposed (Ho et al., 2022). In the miRNA inhibition therapy strategy, an anti-miRNA or miRNA inhibitor, consisting of a single-stranded oligonucleotide with a complementary sequence to mature miRNA, is used. In the miRNA restoration therapy strategy, miRNA mimics that have an identical sequence as the endogenous mature miRNA are introduced into cells to provide an exogenous source of additional miRNAs (Lee et al., 2019).

**TABLE 1:** Experimentally verified miRNAs associated with NPC apoptosis in IDD.

Author, Year	MiRNAs	Direct target	Target signaling pathway(s)	Expression	Reference
Liu et al. (2013)	miR-27a	PI3K	PI3K/AKT	upregulation	Liu et al. (2013)
Wang et al. (2016)	miR-138-5p	SIRT1	PTEN/PI3K/Akt	upregulation	Wang et al. (2016)
Cai et al. (2017)	miR-15a	MAP3K9	p38 and ERK MAPK	upregulation	Cai et al. (2017)
Sun et al. (2018)	miR-532	Bcl-9	Wnt/β-catenin	upregulation	Sun et al. (2018)
Ji et al. (2018)	miR-141	SIRT1	SIRT1/NF-κB	upregulation	Ji et al. (2018)
Chai et al. (2019)	miR-486-5p	FOXO1	FOXO1/NF-κB, mitochondria	downregulation	Chai et al. (2019)
Wang et al. (2019a)	miR-222	BCL2	mitochondria	upregulation	Wang et al. (2019a)
Yang and Sun (2019)	miR-129-5p	BMP2	BMP2/Smad	downregulation	Yang and Sun (2019)
Zhang et al. (2019)	miR-222	TIMP3	TLR4/NF-κΒ	upregulation	Zhang et al. (2019)
Chen et al. (2020)	miR-24-3p	IGFBP5	ERK	upregulation	Chen et al. (2020)
Xu et al. (2020)	miR-423-5p	NLRX1	ERK	downregulation	Xu et al. (2020)
Yang et al. (2020)	miR-96	FRS2	FRS2/PI3K/Akt/CDK2	upregulation	Yang et al. (2020)
Yun et al. (2020)	miR-185	Galectin-3	Wnt/β-catenin	downregulation	Yun et al. (2020)
Cao et al. (2021)	miR-200c-3p	RAP2C	RAP2C/ERK	downregulation	Cao et al. (2021)
Jiang et al. (2021)	miR -338-3p	SIRT6	SIRT6/MAPK/ERK	upregulation	Jiang et al. (2021)
Jie et al. (2021)	miR-125b-5p	TRIAP1	APAF1/Caspase 9	upregulation	Jie et al. (2021)
Xia et al. (2021)	miR-30d	FOXO3	FOXO3/CXCL10	upregulation	Xia et al. (2021)
Zhao and Li (2021)	miR-19b-3p	PTEN	PTEN/PI3K/Akt/mTOR	downregulation	Zhao and Li (2021)
Chen et al. (2022b)	miR-1260b	TCF7L2	Wnt/β-catenin, NF-κB, p53-p21/p16	downregulation	Chen et al. (2022b)
Gao et al. (2022)	miR-143-3p	SOX5	SOX5/SOX9, NF-κB	upregulation	Gao et al. (2022)
Zhou et al. (2022b)	miR-206	GJA1	NF-κB, GJA1/BAX-Caspase-3	upregulation	Zhou et al. (2022b)
Qin et al. (2023)	miR-155	RORα	NLRP3/GSDMD, Bax/Caspase-3	downregulation	Qin et al. (2023)
Wang et al. (2023b)	miR-4306	PAK6	Bax/Caspase-3	downregulation	Wang et al. (2023b)
Zhang et al. (2023a)	miR-4478	MTH1	Bax/Bcl-2/Caspase-3	upregulation	Zhang et al. (2023a)
Jiang et al. (2024b)	miR-365	EFNA3	EFNA3/Eph	downregulation	Jiang et al. (2024b)
Wang et al. (2024b)	miR-874-3p	ATF3	ATF3/Caspase-3	downregulation	Wang et al. (2024b)

Although miRNAs have high specificity for tissue or cell types, the effectiveness of therapeutic miRNAs still has many limitations. One major challenge is the low transfection efficiency, as miRNAs are small and negatively charged, hindering their ability to penetrate the cell membrane, especially in tissues with poor blood supply and low oxygen levels, such as the intervertebral disc. Additionally, due to their single-stranded RNA structure, miRNAs are highly susceptible to degradation by endogenous ribonucleases, resulting in poor stability, which leads to rapid clearance, preventing the miRNA from remaining in the target area to achieve sustained and long-term therapeutic effects. Furthermore, exogenously delivered miRNAs may elicit immune responses.

Despite the promising benefits of miRNA-based therapies, several challenges must be addressed to minimize off-target effects, particularly given the complex regulatory network of miRNAs. Bioinformatics prediction tools including TargetScan, miRDB and DIANA-microT have been widely used to predict miRNA-mRNA interactions and assess potential off-target effects by analyzing seed region complementarity and pathway enrichment. High-throughput screening techniques such as miRNA microarrays and RNA sequencing of degenerated and healthy NP tissue can identify dysregulated miRNAs. And experimental methods are crucial to verify and confirm functional relevance and safety effects, candidate miRNAs should be tested in NPCs cultures or animal models to confirm functional relevance. Dual-luciferase reporter assays can experimentally validate miRNA-mRNA binding, while CRISPR-based miRNA knockout models help assess phenotypic impact. Jie et al. used TargetScan and dual-luciferase reporter to identify the interaction between miR-125b-5p and TP53-regulated inhibitor of apoptosis 1 (TRIAP1). To verify the effect of miR-125b-5p on NPCs. They used IL-1β to treat NPCs to mimic inflammatory microenvironment and discovered that the level of TNF-α and IL-6 in miR-125b-5p inhibitor transfected NPCs decreased. Meanwhile, miR-125b-5p inhibitor improved the cell viability and prevented cells from apoptosis (Jie et al., 2021). Dong et al. recognized miR-489-3p as a potential receptor of Toll-like receptor 4 through TargetScan. Transfected the miR-489-3p mimic significantly decreased the expression levels of TLR4 in human NPCs and suppressed the increase of TNF-α, IL-1β and IL-6 level induced by LPS (Dong and Dong, 2021). These results confirmed the accuracy of miRNA targets predicted by bioinformatics tools. However, miRNA targets predicted by bioinformatics tools need to be verified by *in vivo* or *in vitro* experiments.

## 4 Identified delivery strategies

Studies on the mechanisms by which miRNAs regulate cell apoptosis and subsequently influence the progression of IDD are relatively comprehensive and well developed. However, research on delivery systems to translate these findings into therapeutic applications remains limited and faces several challenges, including encapsulation and release, scalability and storage of exosomes, long-term biocompatibility and immunogenicity. To increase the transfection efficiency, Wang T. et al. employed iron oxide nanoparticles to encapsulate miR-19 and applied an optimal electromagnetic field to achieve responsive release of miR-19 (Wang T. et al., 2023). To improve stability, Qingxin S. et al. demonstrated that employing a multifunctional DNA hydrogel as a carrier for spherical nucleic acids (SNAs) loaded with miR-5590 effectively prevents leakage of the miR-5590-SNA group (Qingxin et al., 2023). This approach significantly reduces potential adverse reactions associated with the medication while enhancing its retention rate within the IVD. Nanoparticles as delivery systems can encounter biocompatibility challenges, with metallic nanoparticles in particular requiring attention due to their potential toxicity—linked to properties such as non-degradability and possible ROS generation. Additionally, the long-term safety of certain inorganic nanoparticles remains an area that warrants further investigation. For instance, in THP-1 cells, silver nanoparticles (Ag NPs) can downregulate CD11b and the response to LPS stimulation, block p62 degradation, induce lysosomal damage, and impair cellular function (Xu et al., 2015). In dendritic cells (DCs), titanium dioxide nanoparticles (TiO_2_ NPs) of varying sizes (20–80 nm) can upregulate the expression of MHC-II, CD80, and CD86 in murine DCs, activate inflammasomes, enhance ROS production, and strongly affect DCs activation states (Winter et al., 2011). In animal studies primarily involving rodents, metal nanoparticles at certain concentrations have shown multi-organ toxicity. Studies involving larger animal models are relatively scarce, necessitating further investigation for clinical applicability. In female ICR mice, oral administration of TiO_2_ NPs for nine consecutive months led to reductions in lymphocyte subpopulations such as CD3^+^, CD4^+^, CD8^+^, and NK cells, indicating toxic effects on lymphoid organs, T cells, and innate immune cells (Hong et al., 2017). In female NIH mice, PLGA-coated superparamagnetic iron oxide nanoparticles (SPIO) caused extensive lysosomal damage, accumulation of LC3-positive autophagosomes, and injury to mitochondria, ER, and the Golgi apparatus. The insufficient mechanical properties and low stability of hydrogels may lead to rapid degradation *in vivo*. While their porous structure facilitates drug burst release, it compromises sustained long-term release. A study found that thermo-responsive PCLA-PEG-PCLA hydrogel loaded with celecoxib demonstrated good safety and short-term efficacy in intradiscal injection in dogs. However, some dogs experienced symptom recurrence after 3 months, indicating that the drug release from the hydrogel may not sustain long-term therapeutic effects (Tellegen et al., 2018). In addition, structural disparities exist in the IVD between rodents/canines and humans, including variations in NP diameter and annulus fibrosus laminations. These differences account for inconsistent impacts on the diffusion behavior and concentration distribution of drug delivery systems, thereby necessitating the establishment of a multi-model validation system prior to the formal initiation of clinical trials. Moreover, many current studies have data collection periods of only a few weeks or months, resulting in relatively short study durations. While hydrogels show promise, their long-term therapeutic efficacy, safety, and potential side effects in the IVD context have not yet been fully characterized. Key aspects such as long-term stability, biocompatibility, and the risk of toxicity or adverse reactions within the IVD environment require additional evaluation.

Exosomes (EVs) serve as efficient carriers for miRNAs, offering protection and ensuring stability. Due to their natural endocytosis mechanism, EVs enable the precise delivery of miRNAs directly into target cells such as NPCs. Furthermore, their diverse biological origins and ease of modification make exosomes versatile and promising tools for therapeutic applications. Many studies have validated the feasibility and efficacy of EV-mediated miRNA delivery in the treatment of IDD Chen. D et al. reported that EV-derived miR-125-5p delivered from cartilage endplate stem cells regulates autophagy and ECM metabolism in the NP by targeting SUV38H1 (Chen and Jiang, 2022). Sun. Y et al. used EVs derived from pluripotent stem cell-derived mesenchymal stem cells to deliver exogenous miR-105-5p to rejuvenate senescent NPCs by activating the Sirt6 pathway *in vitro* (Sun et al., 2021). Chen. F et al. used EVs derived from bone marrow mesenchymal stem cells to deliver miR-155-5p, which can suppress apoptosis in primary mouse NPCs by targeting Trim32 (Chen et al., 2024). However, exosome scalability limitations significantly constrain large-scale standardized production and technical difficulties in long-term storage and maintaining optimal stability complicate their therapeutic application. Currently, the large-scale production of exosomes mainly depends on modified reaction apparatus such as hollow fiber bioreactors and stirred tank bioreactors. These approaches can improve the efficiency of exosome secretion by 3 to 10 folds (Tellegen et al., 2018). Additionally, leveraging the modifiable nature of exosomes, hypoxic-preconditioned EVs (Hu et al., 2023; Zhou Z. M. et al., 2022), selenomethionine-preconditioned EVs (Ma et al., 2024) and selective cago (Liao et al., 2023) sorting have been studied and proven to enhance the therapeutic efficacy of therapeutic miRNA delivery. Furthermore, exosomes exhibit variable immunogenicity profiles depending on their cellular sources, necessitating comprehensive safety evaluations before clinical use.

Research on delivery strategies specifically validated for IDD is limited, and effective approaches are scarce. The low efficiency of these strategies can be attributed to the lack of targeting and responsiveness. Responsive and targeting strategies show great potential and have already demonstrated promising applications in miRNA delivery. To realize smart-controlled release, Wang. Y et al. designed a two-stage inflammation-responsive hydrogel that collapsed initially on the basis of pH-responsive enamine bonds, followed by the controlled release of TA NPs@antagomir-21 relying on pH-/ROS-responsive boronic ester bonds (Wang Y. et al., 2023). Feng. G et al. developed a two-stage MMP-responsive hydrogel system in which cationic block copolymer complexes and miR-29a are mixed with PEG precursors and MMP-cleavable cross-linkers to form polyplex micelle-encapsulated hydrogels *in situ*. In the two-stage release, which occurs at elevated levels of MMP-2, MMP-2 first cleaves the hydrogels to release polyplex micelles, followed by the MMP-responsive detachment of PEG shells from the micelles (Feng et al., 2018). For targeting, Y. Wang et al. reported that the cy3-antagomir-21 released from tannic acid polyphenol-based nanospheres internalized within NPCs was more successful than antagomir-21 itself in spontaneous transfection into cells (Wang Y. et al., 2023).

This review summarizes various targeting and responsive delivery strategies that have been extensively studied for different types of drug delivery. These strategies hold great potential for advancing the development of delivery systems capable of achieving precise spatiotemporally controlled release of miRNAs. While responsive and targeted delivery systems improve efficacy, their long-term biocompatibility and immunogenicity require rigorous evaluation.

## 5 Potential targeting delivery strategies

### 5.1 NPC-targeting strategy

The current NPC-targeting strategy relies mainly on the cellular uptake of delivery systems. Many delivery systems based on nanotechnology have been proven to have better cell uptake effects than therapeutic agents without encapsulation. EVs from various cell sources have been shown to be efficiently taken up by NPCs (Zhu et al., 2020; Hao et al., 2022; Yuan et al., 2020). One study revealed that the cellular uptake of EVs is mediated by caveolae-mediated endocytosis (CVME) and constructed cavin-2-modified engineered EVs to restore EV uptake in TNF-α-impaired NPCs (Ju et al., 2020; Liao et al., 2021). The delivery systems can also reach the target site by binding to specific receptors on the target cells, which has been reported in many disease treatments (Yang et al., 2024). However, few studies have been conducted on IDD treatment, which still requires further exploration but has a promising future. C. Guo et al. incorporated dopamine (DA) into the hyaluronic (HA) macromolecule, synthesizing DA-grafted HA (HADA) to guide the delivery system to specifically bind to CD44 receptors, which are abundant during IDD in NPCs (Guo et al., 2024). The study conducted transfection experiments on TBHP-treated NPCs to compare the distribution of HSGNs and non-targeted NPs under conditions with and without the addition of CD44 antibody. The results showed that HSGNs exhibited significantly higher accumulation in NPCs compared to non-targeted NPs, while this difference was markedly reduced upon the addition of CD44 antibody. This finding confirmed the CD44-targeting capability of HSGNs and the specificity of the interaction between HADA and CD44 (Guo et al., 2024).

**FIGURE 3:**
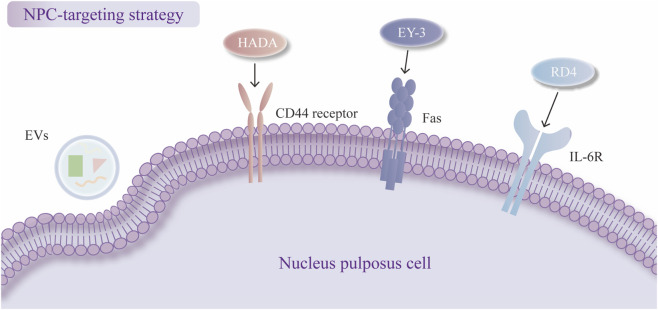
Nucleus pulposus cell (NPC)-targeting strategies. Cellular uptake of exosomes (EVs). Dopamine-grafted hyaluronic (HADA) binds to CD44. EY-3 binds to FasL. RD4 binds to IL-6R.

Moreover, cell-based SELEX can be used to construct an aptamer for NPC targeting, which can be loaded into a delivery system, and studies can choose from among aptamer candidates via flow cytometry analysis (Zhou and Rossi, 2017). H. Jiang et al. synthesized PLGA-PLL-PEG dendrimers equipped with an NPC-targeting aptamer (EY-3), which could directly bind to the transmembrane protein FasL on the surface of NPCs, accelerating the transport of nanoparticles to NPCs (Jiang H. et al., 2024). Chen et al. developed PLGA-PEI-PEG-RD4 nanoparticles with a specific aptamer, and the RD4 aptamer bound to IL-6R on the membrane of NPCs with high affinity (Chen et al., 2023). The NPC-specific aptamer EY3 and RD4 are both selected and optimized via SELEX technology, demonstrated high-efficiency and specific binding to NPC as confirmed by flow cytometry and immunofluorescence (FITC labeling). Mass spectrometry and SDS-PAGE identified FasL and IL-6R as the target molecule of EY3 and RD4 respectively. Moreover, cellular uptake and *in vivo* imaging experiments revealed that PLGA-PLL-PEG-EY3 nanoparticles significantly enhanced transfection efficiency in NPC and exhibited sustained accumulation within intervertebral discs, indirectly confirming targeting specificity. These findings suggest EY3 as a promising targeted delivery vehicle for miRNA-based therapeutics and delivery systems (Jiang H. et al., 2024). Structural analysis unveiled the binding model of the IL-6R-RD4 complex and the key interaction sites at the molecular level, further confirming the specificity of this interaction (Chen et al., 2023; Figure 3).

### 5.2 Organelle-targeting strategy

#### 5.2.1 Advantages and challenges

Subcellular targeting of the ER and mitochondria is an innovative and promising strategy that primarily depends on the incorporation of small-molecule compounds and macromolecules with unique structural properties into delivery systems for targeted accumulation and allows therapeutic agents to be delivered with a higher resolution ratio than can be achieved via a cell-targeting strategy. Organelle-targeting strategy has many advantages. As reviewed above, the apoptosis of NPCs involves organelle-based mitochondrial pathways and endoplasmic reticulum pathways, and organelles can be central to the pathology of IDD. Since the functions and interactions of organelles are more relevant to IDD, the precision of organelle targeting markedly increases the efficacy and reduces off-target damage (Shi et al., 2022).

Despite the promising benefits of organelle targeting, several challenges must be addressed, including the selection of targeting moieties, their conjugation with delivery carriers, and the need to evaluate exclusive versus combined targeting capabilities. The selection of moieties requires a full understanding of the biology of the target organelle, including the unique molecular markers and physicochemical features that may vary in different disease states. The exclusive targeting of one organelle faces many challenges. For example, several ER-targeting moieties are imperfect, as remarkable localization has been detected in other organelles (Cho et al., 2024). Furthermore, since many dysfunctions of organelles occur simultaneously in the occurrence of diseases and many complicated types of crosstalk exist between organelles, one-organelle targeting may be unable to achieve the ideal effect, and some attempts have been made for multi-subcellular targeting (Ma et al., 2016). Currently, few organelle-targeting miRNA delivery systems that are specifically applied to IDD exist. This review summarizes potential strategies for the future development of organelle-targeting delivery systems.

#### 5.2.2 Mitochondria-targeting strategy

Mitochondria, double-membrane organelles with many unique features, can be utilized for designing mitochondria-targeted compounds (MTCs), which have been synthesized, tested, and widely used in delivery systems in the last few decades. There are two main mechanisms based on passive electronic interactions and active peptides with mitochondrial targeting/penetrating sequences.

First, mitochondria are lipophilic double-membrane organelles with high transmembrane potential (ΔΨm) between the matrix and the inner mitochondrial membrane (IMM). Delocalized lipophilic cations (DLCs) can either penetrate membranes or passively accumulate in the mitochondrial matrix because of their highly negative mitochondrial membrane potential. There are many kinds of DLCs, including triphenylphosphonium (TPP), which is a widely used conjugate for mitochondrial targeting (Zielonka et al., 2017), the dicationic amphiphilic molecule called Dequalinium (DQA) (Mallick et al., 2019), the cytotoxic compound (E)-4-(1H-indol-3-ylvinyl)-N-methylpyridineiodide (F16) (Dubinin et al., 2021), rhodamine (Antonenko et al., 2011), pyridinium salts (Qian et al., 2020), cyanine (Saha et al., 2019) and berberine (Fang et al., 2022). The cationic amphiphilic polyproline helix (CAPH) (Kalafut et al., 2012) includes cationic amphipathic α-helical D-(KLAKLAK)_2_ (Lee et al., 2011) and P11LRR (Li et al., 2010).

**FIGURE 4:**
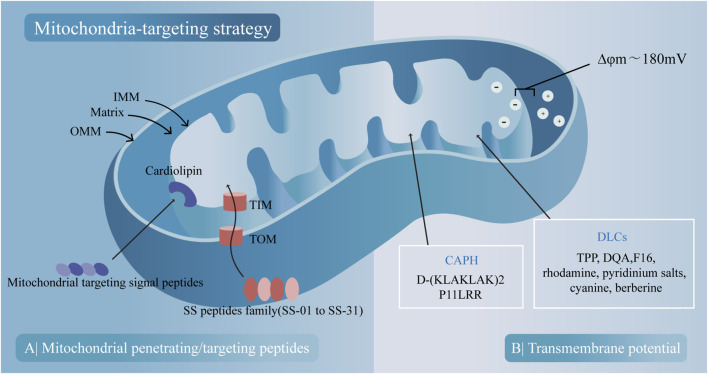
Two main mitochondria-targeting strategies and their corresponding compounds. **(A)** Peptides with mitochondrial-penetrating sequences penetrate mitochondria through outer mitochondrial membrane (OMM) and bind to cardiolipin on the inner mitochondrial membrane (IMM). Peptides with mitochondrial-targeting sequences are imported into mitochondria via translocase of the outer membrane (TOM) and TIM translocase of the inner membrane (TIM) channels. **(B)** Mitochondrial uptake of cationic amphiphilic polyproline helix (CAPH) and delocalized lipophilic cations (DLCs) occurs based on the transmembrane potential.

Second, the mitochondrial penetrating peptides (MPPs) are small peptides developed from cell penetrating peptides (CPPs), which appear to enter cells via a direct mode of uptake and bypass endocytic uptake with the ability to accumulate at the site of the mitochondrial membrane and translocate into the matrix. Cardiolipin-targeting peptides, also known as Szeto-Schiller (SS) peptides, have been synthesized to target the phospholipid cardiolipin, which is exclusively present in the IMM and is closely connected with apoptosis (Paradies et al., 2019; Birk et al., 2013). The SS peptide family includes many peptides (SS-01 to SS-31), and the structure of SS peptides is basically composed of alternate aromatic residues and basic amino acids, allowing them to permeate the plasma membrane and selectively accumulate in the IMM through interactions with cardiolipin (Szeto, 2006). In addition to their ability to target mitochondria, SS peptides can also function as bioactive compounds with antioxidant ability, such as SS31 (Birk et al., 2013). The protein import machinery transports the mitochondrial protein from the cytosol to the IMM, depending on the function of translocase of the outer membrane (TOM) and translocase of the inner membrane (TIM) complex, which appears to be another property to be targeted (Omura, 1998). Based on this mechanism, mitochondrial-targeting signal peptides (MTSPs) have been developed to target the OMM or intermembrane space (IMS). However, the application of the MTSP is limited by its large molecular weight and poor membrane permeability, and further structural optimization is needed (Khan et al., 2022; Figure 4).

#### 5.2.3 ER-targeting strategy

The ER is a highly polymorphic network system that accounts for approximately half of the biomembrane area and is the largest membrane structure in eukaryotic cells, including the rough ER and smooth ER. The ER-targeting strategy is based on the physical and biochemical properties of the ER membrane, similar to the mitochondria-targeting strategy. Although research on ER targeting is relatively rare compared with that on mitochondria targeting (Fang and Chen, 2023), ER targeting holds significant promise for the treatment of IDD. The strategies for ER targeting can generally be divided into small molecule-based targeting and peptide-mediated targeting.

First, based on their ER-localization potential, small molecules have been developed to target the ER via different mechanisms. 1. Ligand‒receptor-mediated targeting. Sulfonyl- and sulfonamide-ligands have high selectivity toward the ER, as represented by glibenclamide and its derivatives, which depend centrally on the specific interaction between its cyclohexyl sulfonylurea moiety and the ATP-sensitive K+ channel (sulfonylurea receptor) that is prominent on the ER membrane (Sikimic et al., 2019). The chlorine group can specifically sense and bind with the chlorine pump in the ER, making it a potential ligand to target the ER (Danylchuk et al., 2021). 2. Lipophilic membrane-mediated targeting. In the ER, lipophilic molecules with certain lipophilic and electric charges can accumulate in the ER membrane. Moreover, based on theories of ‘like dissolves like’ and electrostatic interactions, lipophilic cationic 3,3′-diethyloxycyanine iodine (DiOC6(3)) can be distributed in the cytoplasmic membrane or membranes of subcellular organelles (Shi et al., 2021). Only high concentrations of DiOC6(3) can target the ER, while low concentrations are predominantly distributed in the mitochondria (Sabnis et al., 1997).

**FIGURE 5:**
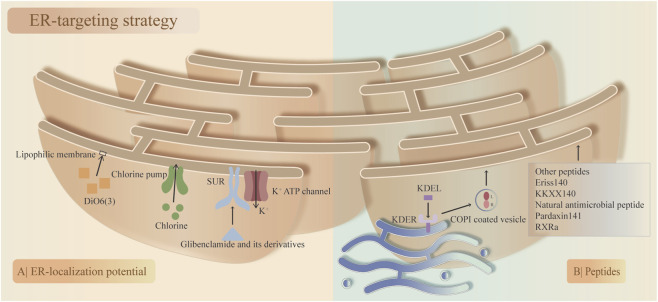
Two main endoplasmic reticulum (ER)-targeting strategies and their corresponding compounds. **(A)** ER-localization potential. The specific interaction between the sulfonylurea ligand (glibenclamide and its derivatives) and the ATP-sensitive K+ channel (SUR; sulfonylurea receptor) on the ER membrane and the chlorine group binding to the chlorine pump on the ER membrane. High concentrations of 3,3′-diethyloxycyanine iodine (DiOC6(3)) accumulate on the ER membrane. **(B)** Peptide signals. KDEL binds to KDELR on the Golgi apparatus, followed by retrograde transport to the ER via coat protein complex (COP I)-coated vesicles, where the ligand‒receptor complex is dissociated. Other peptides that target the ER membrane.

Second, with their unique spatial structure and sequence, peptides can target the ER at a specific site, known as peptide signals. which can be inserted, fused, or localized in the ER membrane and lumen. The ER-retention sequence KDEL was found to accumulate mainly in the ER (Munro and Pelham, 1987). The ER-targeting mechanism of KDEL involves KDEL binding to the KDEL receptor (KDELR, a cell surface receptor that cycles between the plasma membrane and the Golgi complex) in the Golgi apparatus retrograde transport back to the ER via coat protein complex (COP I)-coated vesicles, after which the ligand‒receptor complex is dissociated (Cela et al., 2022). Other peptides also function to target the ER, such as Eriss (Matsuo et al., 2007), KKXX (Matsuo et al., 2007), the natural antimicrobial peptides pardaxin (Ting et al., 2014), and RXR (Scott et al., 2003; Figure 5).

## 6 Potential responsive delivery strategies

### 6.1 Endogenous-stimulus responsive strategies

#### 6.1.1 PH-responsive strategy

The acidic microenvironment is a major characteristic of IVD, as the pH of normal human tissue is estimated to be 7.4. The normal pH value of IVD is reported to be approximately 7.1, whereas that of degenerated IVD can range from 6.8 to 6.2, depending on the severity of IDD (Kitano et al., 1993). Although NPCs can naturally adapt to acidic environment, low pH can also lead to cell damage and apoptosis (Hodson et al., 2018). In response to changes in pH, delivery systems can release therapeutic agents on demand in a controlled manner during IDD. PH-sensitive bond breakage and protonation of chemical groups are two main mechanisms of the pH-sensitive strategy used to release therapeutic agents (Shi et al., 2020). Many pH-sensitive bonds have been synthesized for the delivery of responsive agents, such as hydrazone bonds (Janasik et al., 2022), imine bonds (Ren et al., 2021), ester bonds (Gao et al., 2021), amide bonds (Zhuo et al., 2020), metal ion coordination bonds (Liang et al., 2019), and noncovalent bonds (Maldonado and Amo-Ochoa, 2021). Chemical groups, including carboxyl or amino groups (Gannimani et al., 2020), carboxylic acid groups (Wang et al., 2018), and polyacrylic acid (PAA) (Ding et al., 2022) can be protonated and deprotonated at different pH values. Although the pH gradient is indeed modest, it still reflects a pathological microenvironment that can be selectively targeted by finely tuned pH-responsive materials with narrow activation thresholds. Such specificity has been exploited in other low-pH pathologies like tumors and inflammation tissues.

Liu. S et al. developed a delivery system called SP@NNPm, which enables the pH-responsive release of a PGC1*α* inducer from silica nanospheres (SPs) (Liu et al., 2024). Specifically, SP exhibited a significantly greater release rate in acidic conditions mimicking the intracellular environment (pH 4.5) than in a neutral pH environment (pH 7.5) (Liu et al., 2024). Luo. H et al. designed an injectable self-antioxidant hydrogel (HA/CS) for delivering chondroitin sulfate (CS). When the pH is changed to 6.5, the dissociation of boronic ester bonds from the CS-grafted site and hydrogel-crosslinked site can be triggered; thus, more CS is released in an accelerated manner. Moreover, HA/CS hydrogels exhibit not only pH-responsive delivery behavior but also favorable injectability and mechanical properties (Luo et al., 2023). Lan. W et al. first combines phenylboronic acid-functionalized microgels with strontium sulfite nanoparticles which can respond to pH changes and dissolve at endosomal pH to release miR-155 continuously, thus effectively modulating inflammation and attenuating apoptosis in NPCs (Lan et al., 2025). Guo C et al. designed a microgel encapsulating miR-155. The chitosan coating on the surface of this microgel is ingeniously designed to capture lactic acid and achieve pH-responsive dissociation, thereby alleviating the acidic microenvironment to protect cell viability while facilitating the delivery of miR-155 (Guo et al., 2025). Current applications of pH-responsive delivery systems for IDD are primarily focused on local injection into the lesion site of rodent models, such as mice. This localized delivery approach largely avoids systemic exposure and minimizes the risk of nonspecific release in normal tissues. However, these systems have not yet been validated in primates or humans, and the challenge of ensuring precise activation *in vivo* remains unresolved. Therefore, more comprehensive preclinical studies are required to evaluate delivery specificity and *in vivo* behavior.

#### 6.1.2 ROS-responsive strategy

Redox balance is important for maintaining normal NP functions, and redox imbalance causes oxidative stress, which manifests as increased levels of intracellular ROS. ROS are unstable and highly active molecules that typically include superoxide anions (O_2_), hydroxyl radicals (OH^−^), hydrogen peroxide (H_2_O_2_) and hypochlorite ions (OC1^−^), which may be transferred from one to another. The level of ROS is closely connected to the degree of NPC apoptosis and is positively correlated with the severity of IDD (He et al., 2018), indicating that the delivery of therapeutic agents in response to changes in ROS levels may be suitable and promising. Many materials have been explored for applications involving ROS-responsive release via the mechanisms of carrier dissemblance, carrier degeneration and carrier‒agent linker cleavage (Liang and Liu, 2016). However, ROS-sensitive boronic ester chemical bonds may demonstrate instability and undergo off-target dissociation in delivery systems. This necessitates the implementation of dual-protection carrier systems or microenvironment-triggered dual-release strategies to ensure precise drug delivery.

Liu. C et al. designed a multifunctional hydrogel by self-crossing dopamine (DA)-functionalized gelatin (GelDA) and borax-coupled aldehyde-modified chondroitin (Borax-ACS) via dynamic Schiff base bonds and borate ester bonds, and the DA promoted the attachment of EVs-GLRX3 to the hydrogel network. The responsive-ROS release of EVs-GLRX3 is attributed to the ROS sensitivity of DA and the cleavage of borate ester bonds (Liu et al., 2023). Yu. H et al. fabricated an isoginkgetin-loaded ROS-responsive delivery system (IGK@SeNP) based on diselenide block copolymers, which can release IGK intelligently in microenvironments with high levels of ROS and scavenge ROS because of the reducibility of Se-Se bonds (Yu et al., 2023). Feng. Y et al. blended modified polyvinyl alcohol (MPVA) and polycaprolactone (PCL) through electrospinning, creating a composite fiber membrane with excellent ROS-responsive capability due to the degradation of MPVA in a high-ROS microenvironment, while NO-loaded BioMOFs were synthesized and incorporated into the electrospun MPVA/PCL fiber membrane (LN@PM) to achieve the controlled release of NO-loaded BioMOFs (Feng et al., 2024).

#### 6.1.3 Enzyme-responsive strategy

During IDD, the levels of enzymes such as matrix metalloproteinases (MMPs), tissue inhibitors of metalloproteinases (TIMPs) and disintegrins and metalloproteinases with thrombospondin motifs (ADAMTSs) increase (Zhang et al., 2020). MMPs are a class of endopeptidases that are dependent on zinc and capable of cleaving most of the protein components of the ECM, thus playing a crucial role in the unbalanced ECM degradation of IVD (Zou et al., 2023). ADAMTS can bind to the components of the ECM efficiently to cleave aggrecan, and TIMPS can inhibit MMP and ADAMTS enzymatic activity to maintain the balance of the matrix (Porter et al., 2005). Enzyme-responsive strategies exhibit excellent catalytic efficiency, and in present studies of IDD, enzyme-responsive strategies have focused mainly on MMP-responsive strategies. MMP-responsive delivery can be achieved through the cleavage of MMP-cleavable peptides on the surface of biomaterials, such as the cleavage of mesoporous silica nanoparticles, the backbone of the hydrogel and the linker between the drugs and the nanoparticles (Zhang C. et al., 2023). Since the precise personal expression level of various enzymes is difficult, accurate biomarkers are required for delivery systems.

Zheng. Z et al. encapsulated the pH-responsive H_2_S donor JK1 into a collagen hydrogel (Col-JK1) to form a pH- and MMP-responsive H_2_S release system. JKs, especially JK1, can release H_2_S at acidic pH values via a pH-dependent intramolecular cyclization reaction but rarely generate H_2_S under neutral pH conditions. With the addition of MMP-9, a much faster generation of H_2_S was observed, indicating the enzyme responsiveness of Col-JK1 in releasing H_2_S owing to cleavage of the backbone of the hydrogel (Zheng et al., 2019).

### 6.2 Exogenous-stimulus responsive strategies

#### 6.2.1 Thermoresponsive strategy

The principle of a temperature-responsive strategy relies on changes in the solubility of the delivery system in response to temperature variations (Karimi et al., 2016). A temperature-responsive strategy has been widely used for the advantages of being introduced into the body in a minimally invasive manner prior to undergoing a phase transition to a solid or gel state (Bikram and West, 2008). A low critical solution temperature (LCST) is a special feature of some polymeric materials, such as poly (N-isopropylacrylamide) (PNIPAAm) derivatives (Yoshida et al., 1994), copolymers of poly (ethylene glycol) and poly (N-isopropylacrylamide) (PEO–PPO) pluronic copolymers (Zarrintaj et al., 2020), core–shell thermoresponsive nanoparticles (Kashyap and Jayakannan, 2014), layer-by-layer (LBL)-assembled nanocapsules (Zhou et al., 2014), polymeric micelles (Chung et al., 1999), and elastin-like polypeptides (ELPs) (Ryu and Raucher, 2014). This property has been the basis for the design of many temperature/thermal-responsive strategies. The safety of the delivery system has to be ensured prior to advancement since the polymeric systems like PNIPAAm can be degraded within the body to produce toxic products, which may outweigh the benefits of controlled release (Bikram and West, 2008).

A study developed a decellularized extracellular matrix (dECM)-derived hydrogel for the thermal-responsive delivery and sustained release of EVs (Xing et al., 2021). Z. Liao et al. developed a composite hydrogel (FEC) as an EV carrier composed of thermoresponsive pluronic F127 and biocompatible dECM derived from NP tissues. Pluronic F127, a well-known thermoresponsive copolymer, can form a semisolid gel at 37 °C. The FEC hydrogel with 0.1% dECM demonstrated a smooth sol‒gel transition at 37 °C, allowing for the controlled release of EVs from the delivery system (Liao et al., 2022). Willems. N et al. designed a thermoresponsive hydrogel using PNIPAAm MgFe-layered double hydroxide for the intradiscal controlled delivery of CXB. The hydrogel rapidly transitioned from a low-viscosity state to a stable gel within 10 s as the temperature rose above its 32 °C LCST, driven by hydrophobic interactions between the isopropyl groups of pNIPAAM (Willems et al., 2015). Wang. J et al. also designed injectable N-PNIPAAM-based thermosensitive hydrogels for the delivery of SHP099, which is a small-molecule inhibitor of IDD pathology-related Src homology region 2-containing protein tyrosine phosphatase 2 (SHP2) (Wang et al., 2021). Cunha. C et al. and Pereira. C L et al. evaluated a delivery system (HAPSDF5) using a hyaluronan-based thermoresponsive hydrogel (HAP) loaded with the chemoattractant SDF-1 to recruit MSCs. The HAP hydrogel, composed of hyaluronan and poly (N-isopropylacrylamide), forms an injectable copolymer that quickly gelifies *in situ* at temperatures above 30 °C (Cunha et al., 2021; Pereira et al., 2014).

#### 6.2.2 Light/photo-responsive strategy

Light functions as an external stimulus that can be precisely modulated in terms of irradiation time, spatial location, intensity, and wavelength. Based on the wavelength of exposure, light can be classified into three subgroups: ultraviolet (UV) light (200–400 nm), visible (Vis) light (400–700 nm), and near-infrared (NIR) light (700–1,000 nm) (Silva et al., 2019). Light/photo-responsive strategies have gained prominence in the spatiotemporal regulation of delivery systems, enhancing their therapeutic efficacy and safety profiles. These strategies can be categorized into two principal types: direct responsive systems, which engage in photoreactions directly involving the drug or delivery vehicle; employing materials and chemical structures that respond to UV, Vis, and NIR wavelengths; and indirect responsive systems, which rely on light-generated mediators—such as heat, ROS, hypoxia, and gas molecules—to trigger subsequent structural transformations (Zhou et al., 2018). Light/photo-responsive strategies also have critical shortcomings, the majority of the responsive motifs of direct responsive systems has low sensitivity to NIR ligh and mediators of indirect one may cause ROS- or heat-induced damage to cargo molecules. However, it is important to consider the safety considerations of photostimulation. For instance, UV may pose risks of cellular damage or carcinogenicity at certain doses, while NIR radiation can induce thermal stress in cells if parameters are not carefully controlled. And NIR irradiation itself has a penetration limit of approximately 1 cm, which makes it hard to reach diseased sites located in the deeper parts of the body (Karimi et al., 2017).

Vaudreuil. N et al. explored a minimally invasive tissue engineering approach by injecting a photopolymerizable, biodegradable methacrylated gelatin-based biogel scaffold loaded with mesenchymal stem cells into the NP. The scaffold rapidly gelates under visible light, using lithium phenyl 2,4,6-5 trimethylbenzoylphosphinate (LAP) as the photoinitiator, avoiding the risk of DNA damage associated with UV light. Photopolymerization was achieved *in situ* via fiberoptic visible light irradiation (Vaudreuil et al., 2019). Tao. S et al. reported that local administration of nitric oxide (NO)-releasing micellar nanoparticles effectively treats IDD associated with Modic changes in a rat model infected with Cutibacterium acnes (C. acnes). By covalently incorporating a palladium (II) meso-tetraphenyltetrabenzoporphyrin photocatalyst and coumarin-based NO donors into the nanoparticles, they showed that the UV-absorbing coumarin-based NO donors could be activated under red light irradiation via photoredox catalysis. The resulting micellar nanoparticles exhibited stability in aqueous solutions without premature NO leakage, enabling controlled NO release under red light under physiological conditions (Tao et al., 2022).

#### 6.2.3 Ultrasound-responsive strategy

Ultrasound has emerged as a promising external physical stimulus for responsive delivery strategies, leveraging either low-frequency (<100 kHz) or high-frequency (>100 kHz to MHz range) waves to increase delivery efficiency, which is achieved primarily through thermal and nonthermal effects (Matafonova and Batoev, 2019). The thermal effects result from the absorption of acoustic energy by biological tissues but thermal effects may induce protein denaturation or cell necrosis if temperature rises exceed 5 °C–6 °C, whereas the nonthermal effects arise from ultrasound pressure, acoustic streaming, shockwaves, liquid microjets, and ultrasound-induced oscillations or cavitation (Mannaris et al., 2020). The presence of cavitation nuclei—particles that reduce the acoustic intensity needed to trigger cavitation—greatly enhances the efficiency of ultrasound-mediated delivery (Ren et al., 2022). With the rapid progress in nanotechnology, novel nanomaterials, including nanobubbles (NBs) (Endo-Takahashi and Negishi, 2020), droplets (Lea-Banks et al., 2019), micelles (Jahanbekam et al., 2023), and nanoliposomes (Fan et al., 2022) have been developed as ultrasound-responsive delivery strategies. However, high frequency ultrasound has poor penetration ability, especially with the frequencies of 12 and 30 MHz may experience significant attenuation when passing through cancellous bone, mainly within the trabecular bone. Moreover, the difference in human body temperature and experimental temperature, as well as the difference in sound velocity, makes the ultrasound navigation environment much more complex than the experimental environment, further limiting the penetration effect of ultrasound (Li et al., 2024).

Shen. J et al. synthesized resveratrol (RES)-embedded NBs via a double-emulsion method and then conjugated the NPC-targeting antibody CDH2 (AbCDH2) to the NBs via a carbodiimide reaction. When exposed to ultrasound (US), inert gas-filled NBs undergo inertial cavitation, creating microjets that improve drug delivery to adjacent cells. In the absence of ultrasound, the release of RES was slow, with only 20% released at 6 h and 35% at 72 h, indicating that US effectively induces NB carriers to release RES efficiently through its cavitation effect (Shen et al., 2018). Nguyen. K et al. encapsulated hydrophobic simvastatin, a potential IDD drug, in perfluorocarbon liquid droplets via a double-emulsion technique. High-intensity focused ultrasound (HIFU) was then applied at specific intervals to release simvastatin at controlled doses. HIFU induces acoustic droplet vaporization, causing liquid droplets to transform into gas bubbles. This process not only facilitates drug release but also renders the bubbles detectable via ultrasound due to their echogenic properties (Nguyen et al., 2019).

## 7 Conclusion and future perspectives

This review highlights the role of NPC apoptosis in IDD and identifies the mechanisms involved in the apoptosis of NPCs. Moreover, the miRNAs involved in these mechanisms and their delivery strategies are also summarized here. Furthermore, potential research and development directions for this field are proposed.

Excessive apoptosis of NPCs impairs the normal anabolic/catabolic balance of the ECM, which is the main function of the nucleus pulposus. Mainly through inducing exacerbated inflammation, the apoptosis of NPCs not only decreases the production of the ECM but also promotes the degradation of the ECM. Furthermore, apoptosis activates other modes of death, such as pyroptosis and necrosis, and inhibits autophagy in nucleus pulposus cells, resulting in the loss of normal cells and the ECM in the nucleus pulposus. All the common approaches, including the DR-mediated approach, the mitochondrial approach, and the endoplasmic reticulum approach, affect the apoptosis of NPCs. Factors such as aging, mechanical stimulation, and microtrauma can activate these pathways and consequently induce the apoptosis of NPCs.

Recently, miRNAs have been identified as effective therapies for NPC apoptosis. In general, hundreds of miRNAs can modulate NPC apoptosis in degenerated IVD. Exploring new mechanisms is essential for promoting their clinical use. However, previous studies have focused mainly on the miRNAs that affect the DR-mediated approach and the mitochondrial approach. Few studies have revealed miRNAs involved in ER approaches. For example, miR-155, miR-499a-5p, miR-25-3p, and miR-532-5p mainly modulate NPC apoptosis through the DR approach, and miR-143, miR-222, miR-573, and miR-14 mainly regulate apoptosis through the mitochondrial approach. The use of miRNAs faces challenges, including low transfection efficiency, poor stability, and off-target effects. Therefore, developing delivery strategies is essential for its use. Since IVD is an avascular tissue, un-modified carriers have low passive diffusion efficiency, and the dense ECM and negatively charged cell membrane hinder miRNA penetration, requiring active targeting strategies such as ligand receptor binding and aptamers to improve delivery efficiency. However, the targeting capability of existing systems is still limited. The prominent biocompatibility issues and long-term safty of nanodelivery systems, especially inorganic nanoparticles remain questionable. Delivery systems based on hydrogel may face the problems of low stability, rapid degradation and burst release of therapeutic agents. And the criteria for toxicity assessment of delivery systems are lacking, which makes the evaluation indicators for each team in the stage of developing new delivery systems often varying. For example, not all experiments have appraised the poisonousness of empty carriers (De Jong and Borm, 2008). Although EVs have natural targeting advantages, large-scale production faces difficulties in purity control, with fluctuations in drug efficacy rates between batches exceeding 40%, and poor storage stability (Chen and Jiang, 2022). Notwithstanding, the aforementioned delivery systems still possess significant potential for further development and application. In particular, the development of multimodal delivery systems that integrate various targeting and responsive strategies to overcome these shortcomings can enhance delivery efficiency and targeting accuracy. For instance, combining NPC-targeting aptamers with stimuli-responsive chemical bond modifications to develop precise targeting and multi-stimuli responsive delivery systems. Additionally, exploring the combined therapeutic use of miRNA with other drugs, although currently few studied, has also demonstrated immense potential as a treatment strategy. Currently, the application of delivery systems in the treatment of IDD has been restricted to cells and rodents. Tellegen et al. observed that intradiscal injection of PCLA-PEG-PCLA hydrogel loaded with celecoxib could significantly relieve back pain in canines. However, studies on delivery systems loaded with miRNA in large animals or primates are still blank and deserve extended attention (Tellegen et al., 2018). Few delivery systems have been used to treat intervertebral disc degeneration in clinical trials. One controlled, randomized, double-blind placebo clinical trial evaluates the safety and efficacy of PRP with exosomes administration at the center of the nucleus pulposus in discogenic LBP (NCT04849429). And a cohort study assess the safety and the efficacy of an hydrogel (double cross-link microgel-DXM) injection into the intervertebral disc space (NCT04727385). But neither of these studies had definite positive safety results, there is still a long way for delivery systems in IDD from bench to bedside.
